# Impact of the COVID-19 pandemic lockdown on Body Mass Index: a three-year follow up study in 6,156 Chinese college students

**DOI:** 10.3389/fendo.2024.1387151

**Published:** 2024-06-20

**Authors:** Haoxuan Li, Yiling Song, Yangyang Wang, Xiaolu Feng, Chengwei Li, Jianmin Peng, Hongjun Yu

**Affiliations:** ^1^ Department of Physical Education, Tsinghua University, Beijing, China; ^2^ Department of Sports Science, College of Education, Zhejiang University, Hangzhou, China

**Keywords:** BMI, Covid-19, college students, obesity, lockdown

## Abstract

**Background:**

The novel coronavirus disease 2019 as the most pervasive and consequential pandemic in recent years, has exerted significant impacts on human health, including aspects related to body weight. Objectives: This study aims to assess the influence of the lockdown measures implemented during the COVID-19 pandemic on Chinese college students’ Body Mass Index (BMI) through a three-year cohort study.

**Methods:**

We recruited 6156 college students (n = 4,248, 69% male, and n = 1,908, 31% female, with an average age of 18.68 ± 0.86 yr.) from a University in China to participate in this three-year cohort study. All of the subjects took the same physical fitness tests from 2019 to 2021 (pre-lockdown, during lockdown and post-lockdown). Participants’ height and weight data were objectively measured by Tongfang Health Fitness Testing Products 5000 series. A paired t-test was performed in the analysis.

**Results:**

During the lockdown, there is 4.2% increase of BMI among the college student (p<0.001). Moreover, males had a greater overall mean BMI rate increase of 4.74% (p<0.001) than females (2.86%, p<0.001). After the lockdown, there is 0.94% increase of BMI among the college student (p<0.001). However, females had a greater overall mean BMI rate increase of 1.49% (p<0.001) than males (0.72%, p<0.001). During this period, the obese and overweight group’s growth rate from 2019 to 2020 was smaller than the normal and underweight group, which were 2.94% (p<0.001), 3.90% (p<0.001), 4.44% (p<0.001) and 5.25% (p<0.001), respectively.

**Conclusion:**

BMI increased both during and post-lockdown periods among Chinese college students. However, during the lockdown, participants with higher BMI groups appeared to have a diminished BMI growth rate compared to those with lower BMI. After the lockdown, participants with higher BMI levels appeared to have an augmented BMI growth rate. Public policy action is needed to increase the level of physical activity of Chinese college students and take action to improve students’ physical fitness performance after the lockdown.

## 1 Introduction

The novel coronavirus disease 2019 (COVID-19) was characterized as a pandemic by the World Health Organization (WHO) on March 11, 2020 (1.3.11). The outbreak has a huge impact on countries globally, affecting approximately 200 countries and territories, posing a gigantic threat to people’s mental health and well-being (2–4). To restrain the influence of the pandemic, governments worldwide implemented a diverse array of actions to cut off the transmission pathway and mitigate the spread. For instance, China had implemented closure measures such as city lockdowns, social distancing, holiday extensions, and doing self-quarantine to curb the harm of the pandemic (5). Universities in China had also experienced a lockdown period (6). In terms of restraining the spreading of the pandemic, universities made several restrictions to limit students’ social activities such as closing public areas, changing classes to online, controlling exits of the school, and so on. These lockdown measures were proved to be successful as the previous research showed the data indicating a significant decrease of in the case growth rate in Wuhan (7). However, researches showed that the lockdown during the pandemic would induce the negative change in eating habits, lifestyle, and health conditions (8–11). Study showed that there are significant differences from before to during the pandemic in people’s physical activity (significant decreases), self-esteem (significant decreases), and social physical anxiety (significant increases) regardless of their gender due to the restrictions (12). People also experienced different degrees of disruption in their daily routines and physical activity during the pandemic (13). A Switzerland study has suggested that the lockdown had a negative impact on the BMI of youth aged 6–18 in both normal weight and with obesity (14). The fluctuation of BMI was also appear in the result of an online longitudinal study in 1818 UK adults across the first year of the pandemic (15). The negative changes that were brought by the lockdown have been evident across all age groups.

Overweight and obesity is a prevalent and essential problem that impairs human health. Children with obesity were more likely to suffer not only metabolic diseases but also mental health (16). China is facing a huge challenge of obesity. In 2010, Chinese overweight and obese young people had almost doubled from 10 years ago (17). In 2015, China contributed to 5.1% of global obesity (16). Moreover, many factors may induce people’s overweight and obesity problems. Among these, lack of physical activity (PA) is one of the essential ones. PA has benefits for human health. Study showed that through consistent participation in regular PA, adults can not only facilitate and maintain good health conditions but also reduce the risk of chronic disease and premature mortality (18). On the contrary, the inactivity of PA could lead to joint diseases, obesity and mental health impairment (19).

With the fact that over 91% of the world’s students were negatively impacted due to the nationwide school closures and home confinement, the disruption of PA existed (3). According to the a retrospective observational study of Chinese college students during the lockdown period, there is a significant increase in sedentary time and weight gain in both genders (20). In addition, under the lockdown circumstance, college students are also suffered from bad mental conditions such as depression and anxiety (21). There is emerging evidence showing that the lockdown has a significant impact on college students’ PA engagement, mental health, obesity status, sedentary behavior, and so on (22–26).

Despite previous research findings, however, gaps in the impact that the COVID-19 lockdown has on college students’ BMI remain. First, research on the impact of Covid-19 on Chinese college students’ BMI is limited. Second, most papers concentrated attention on the small sample and self-reported method to evaluate the pandemic’s influence on obesity among the subjects. This disproportion may leave the bias remaining. Last but not least, it is important to note that only a limited number of studies have implemented a cohort follow-up study for more than two years during the COVID-19 lockdown as well as taking participants’ baseline BMI status into consideration.

In conclusion, given these gaps and limitations in the existing literature, a study that aims to address these issues by implementing a more than two years longitudinal study with a large sample size and objective measurements is needed. It will not only fill the existing research gaps but also ensures a more accurate and reliable assessment of the situation. Thus, to address these gaps, this study aims to estimate the impact of the COVID-19 lockdown on BMI among Chinese students by implementing a three-year large sample objective measurement. By using a cohort follow-up three consecutive years study design, this research carried out the measurements three times: pre-lockdown, during lockdown and post-lockdown.

## 2 Materials and methods

### 2.1 Participants

**Figure 1:**
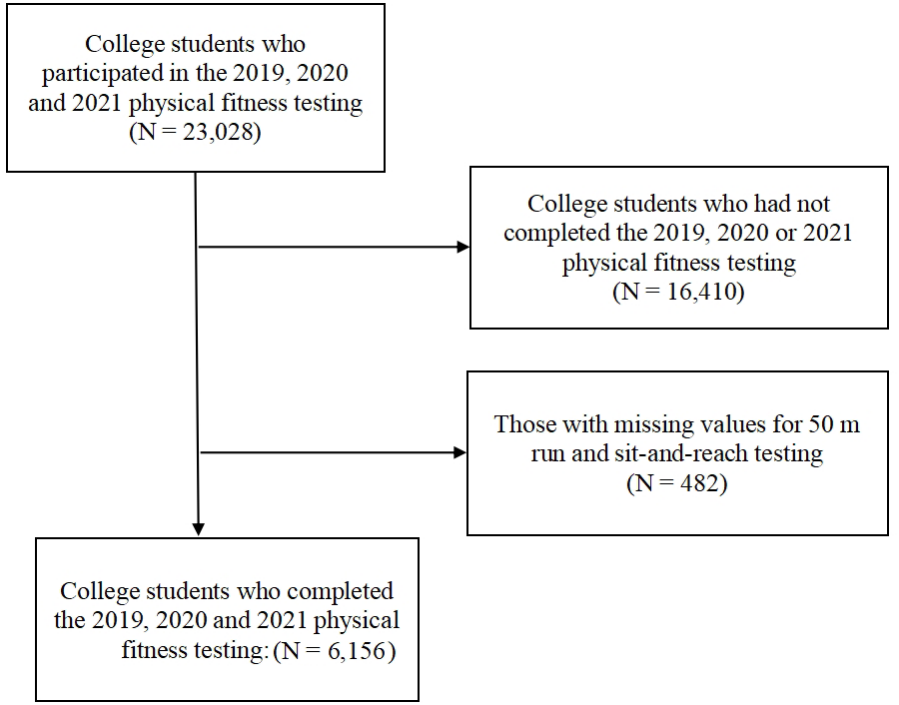
Study sample flowchart.

This study conducted a three-year longitudinal study. We recruited 6156 college students (n = 4,248, 69% male, and n = 1,908, 31% female, with an average age of 18.68 ± 0.86 yr.) from Tsinghua University in China to participate in this three-year cohort study. All of the subjects took the same physical fitness tests from 2019 to 2021 (pre-lockdown, during lockdown and post-lockdown) (see **Figure 1**).

### 2.2 Procedures

Tsinghua University Fitness Center carries out physical fitness tests every autumn semester. By strictly following the requirement of the National Student Physical Health Standard (NSPHS) in China (27), the physical fitness test measures height and weight in both genders.

In this study, the lockdown of the university started in mid-January and ended in late August 2020. During this period, all the participants were asked to do the self-quarantine at home, staying away from the school-based PA. As the period ended, the university lifted the restrictions so that students could return to school and resume their school-based PA. Under this circumstance, the pre-lockdown test was implemented from September 9 to November 10, 2019. All the anthropometric data were collected before the lockdown period. The during lockdown test was carried out from October 19 to November 14, 2020. Since it’s conducted several weeks after the lockdown ended, the test is best likely to reflect the participants’ changes that occurred during the lockdown period. Participants were asked to finish the test after the end of the lockdown. The post-lockdown test took place from October 30 to November 14, 2021, that is a whole year after the campus reopened. All of the tests in 2019–2021 were conducted from 8:00 a.m. to 12:15 p.m., using the same objectively measurements and procedures. This research was approved by the ethics committee of the university (IRB #201253400). All the participants knew the data would be used only in future research and had given their informed consent.

### 2.3 Height and weight measurement

The height and weight data were collected by the height and Weight tester of TongFang health Fitness testing Products 5000 series (TongFang health technology co., ltd., Beijing, china) (28). This machine can measure height and weight simultaneously. To collect the data, participants need to stand on the machine bare footed with light clothing. The height (cm) measuring starts from the top of the participants’ heads and extends to the bottom of their heels, while the weight (kg) measuring occurs in conditions where participants wear no shoes and light clothes. Height and weight were measured to the nearest 1cm and 0.5 kg, and BMI was calculated by weight(kg) divided by height square (m^2^).

### 2.4 Statistical analyses

Two types of analyses were used to evaluate the impact of COVID-19 on students’ BMI. Participants are divided in the 4 groups according to their BMI strata (underweight,<18.5; normal, 18.5–22.9; overweight, 23.0–24.9; obesity, ≥25) (29). Descriptive statistics were conducted to illustrate the basic characteristics of the 4 groups including means and standard deviations. Paired-sample t-tests were employed to estimate the changes of the 4 groups’ BMI during the lockdown by comparing the pre-lockdown data with during-lockdown data and post-lockdown data respectively. We calculate the BMI changes compared with the baseline as below. For example,


$$2020\text{ BMI change\%}=\frac{\left(BMI\;in\;2020-BMI\;in\;2019\right)}{BMI\;in\;2019}\times 100\%$$

All of the statistical analyses were conducted by using IBM SPSS Statistics 27 (SPSS, Inc., Chicago, IL, USA). The statistical significance was set at p<0.05.

## 3 Results

### 3.1 The basic characteristics of participants

**Table 1** shows the baseline characteristics of the participants in the pre-lockdown test in 2019. A total of 6156 college students (69% in men and 31% in women) from Tsinghua University engaged in the three-year cohort study from 2019 to 2021. The participants’ overall mean age was 18.68 years (SD=0.86) and their mean height and weight were 172.58cm (SD=8.09) and 64.36kg (SD=12.35) respectively. The male has a greater height and weight result than the female (p<0.01).

**Table 1:** Basic characteristics of the participants in the pre-lockdown test (2019).

		Underweight	Normal	Overweight	Obese	Total	p
Male	N	577	2290	687	694	4248	
	Age, mean (SD)	18.58 (0.83)	18.67 (0.86)	18.71 (0.97)	18.74 (0.92)	18.67 (0.88)	0.009
	Height, mean (SD)	176.60 (6.30)	176.16 (5.96)	176.15 (6.18)	176.39 (6.16)	176.252 (6.08)	0.386
	Weight, mean (SD)	54.65 (4.55)	64.59 (5.96)	74.23 (5.62)	86.86 (10.67)	68.43 (11.85)	<0.001
Female	N	430	1215	157	106	1908	
	Age, mean (SD)	18.53 (0.73)	18.73 (0.83)	18.71 (0.95)	18.79 (0.78)	18.69 (0.82)	<0.001
	Height, mean (SD)	165.3 (5.45)	164.14 (5.56)	163.60 (5.76)	164.53 (5.85)	164.40 (5.56)	<0.001
	Weight, mean (SD)	48.06 (3.74)	55.1 (4.85)	63.85 (4.58)	73.84 (7.4)	55.28 (7.73)	<0.001
Total	N	1007	3505	844	800	6156	
	Age, mean (SD)	18.56 (0.79)	18.69 (0.85)	18.71 (0.96)	18.75 (0.9)	18.68 (0.86)	0.500
	Height, mean (SD)	171.82 (8.13)	171.99 (8.18)	173.81 (7.81)	174.81 (7.32)	172.58 (8.09)	<0.001
	Weight, mean (SD)	51.83 (5.33)	61.3 (7.19)	72.3 (6.78)	85.13 (11.20)	64.36 (12.35)	<0.001

### 3.2 The BMI result

**Table 2** presents the BMI outcome of the participants in the three-year follow up study from 2019 to 2021 (pre-lockdown in 2019, during lockdown in 2020 and post-lockdown in 2021, respectively). Participants across varying weight groups appear to have a significant increase in BMI from 2019 to 2021.

**Table 2:** The BMI outcome of the participants from 2019 to 2021.

		Underweight	Normal	Overweight	Obese	Total	p
Male	N	577	2290	687	694	4248	
	BMI, mean (SD)						
	2019 year (s)	17.50 (0.82)	20.79 (1.26)	23.89 (0.58)	27.87 (2.69)	22.00 (3.46)	<0.001
	2020 year (s)	18.54 (1.27)	21.89 (1.69)	24.97 (1.55)	28.7 (3.37)	23.04 (3.65)	<0.001
	2021 year (s)	18.53 (1.40)	22.01 (1.91)	25.22 (1.98)	29.07 (3.73)	23.21 (3.89)	<0.001
Female	N	430	1215	157	106	1908	
	BMI, mean (SD)						
	2019 year (s)	17.55 (0.77)	20.43 (1.22)	23.83 (0.54)	27.25 (1.95)	20.44 (2.59)	<0.001
	2020 year (s)	18.32 (1.06)	21.00 (1.60)	24.13 (1.65)	28.02 (3.07)	21.04 (2.79)	<0.001
	2021 year (s)	18.54 (1.24)	21.34 (2.65)	24.61 (1.86)	28.25 (3.74)	21.36 (3.34)	<0.001
Total	N	1007	3505	844	800	6156	
	BMI, mean (SD)						
	2019 year (s)	17.52 (0.80)	20.67 (1.26)	23.88 (0.57)	27.79 (2.61)	21.52 (3.29)	<0.001
	2020 year (s)	18.44 (1.19)	21.58 (1.71)	24.81 (1.61)	28.61 (3.34)	22.42 (3.53)	<0.001
	2021 year (s)	18.53 (1.33)	21.78 (2.22)	25.11 (1.97)	28.96 (3.74)	22.64 (3.83)	<0.001

The same growing trend also can be observed in both genders. Overall, male has a higher BMI outcome than female each year. Among them, in the group of underweight, the average BMI of female is slightly greater compared to that of males in 2019 and 2021. Conversely, among the other weight groups, the male BMI surpassed that of females. Furthermore, in 2019, while the mean BMI difference between genders in the underweight, normal, and overweight groups remained minimal, it became pronounced within the obese group.

### 3.3 Impact of COVID-19 on BMI

**Table 3** presents the results of paired t-test examining the variations in participants’ BMI by gender and different weight groups in pre-lockdown (2019) and during lockdown (2020) time period. Overall, each weight group has a significant BMI increase from 2019 to 2020, though the growth rate exhibited a reduction with the increase of weight baseline (p<0.001). The BMI of male participants displayed significant increase across underweight, normal, overweight and obese groups, denoted as 5.92%, 5.30%, 4.50% and 2.95%, respectively (p<0.001). The BMI of female participants also presented a significant increase in underweight, normal, overweight and obese groups, denoted as 4.35%, 2.78%, 1.28% and 2.86%, respectively (p<0.001). In total, male participants has a more pronounced elevation in BMI compared to female participants during the pandemic, with observed increase of 4.74% and 2.95% for males and females, respectively (p<0.001).

**Table 3:** The paired t-test results of the participants’ BMI from 2019 to 2020.

2020–2019	Underweight				Normal				Overweight				Obese					Total			
	Paired difference mean (95% CI)	Change (%)	Effect size (95% CI)	t	Paired difference mean (95% CI)	Change (%)	Effect size (95% CI)	t	Paired difference mean (95% CI)	Change (%)	Effect size (95% CI)	t	Paired difference mean (95% CI)	Change (%)	Effect size (95% CI)	t	Paired difference mean (95% CI)		Change (%)	Effect size (95% CI)	t
Male	1.04 (0.96, 1.11)	5.92%↑	1.11 (1.00, 1.20)	26.57^***^	1.10 (1.06, 1.15)	5.30%↑	0.97 (0.92, 1.02)	46.43^***^	1.07 (0.96, 1.19)	4.50%↑	0.72 (0.63, 0.80)	18.81^***^	0.82 (0.66, 0.98)	2.95%↑	0.39 (0.31, 0.47)	10.27^***^	1.04 (1.00, 1.08)		4.74%↑	0.75 (0.72, 0.79)	49.17^***^
Female	0.76 (0.68, 0.85)	4.35%↑	0.88 (0.77, 0.99)	18.19^***^	0.57 (0.51, 0.63)	2.78%↑	0.51 (0.45, 0.57)	17.75^***^	0.31 (0.07, 0.55)	1.28%↑	0.20(0.04, 0.36)	2.51^*^	0.78 (0.39, 1.17)	2.86%↑	0.38 (0.18, 0.58)	3.93^***^	0.60 (0.55, 0.66)		2.95%↑	0.51 (0.46, 0.56)	22.25^***^
Total	0.92 (0.86, 0.98)	5.25%↑	1.00(0.93, 1.08)	31.78^***^	0.92 (0.88, 0.96)	4.44%↑	0.79 (0.75, 0.83)	46.92^***^	0.93 (0.83, 1.04)	3.90%↑	0.61(0.54, 0.68)	17.68^***^	0.82 (0.67, 0.96)	2.94%↑	0.39(0.32, 0.46)	11.00^***^	0.91 (0.87, 0.94)		4.21%↑	0.68(0.65, 0.70)	53.10^***^

*p<0.05; ***p<0.001; ↑: increase.

**Table 4** presents results of paired t-test examining the variations in participants’ BMI by gender and different weight groups in during lockdown (2020) and post-lockdown (2021) time period. Overall, each weight group has a significant BMI increase from 2020 to 2021, and the growing rate ascended together with participants’ BMI increasing (p<0.01). But the elevation is modest compared with that rising from 2019 to 2020. The male participants’ BMI had a no statistically significant decrease (0.04%, p=0.83) in underweight group after the lockdown in 2021. Other male participants’ BMI in normal, overweight and obese groups demonstrated significant increases in 0.55%, 1.03%, and 1.31%, respectively (p<0.001). The BMI of female participants exhibited a pronounced increase in underweight, normal, overweight and obese groups, denoted as 1.2%, 1.58%, 1.96% and 0.8%, respectively. In total, female participants had a more prominent increase than in BMI compared to male participants after the pandemic, with observed increase of 1.49% and 0.72% for males and females, respectively (p<0.001).

**Table 4:** The paired t-test results of the participants’ BMI from 2020 to 2021.

2021–2020	Underweight				Normal				Overweight					Obese					Total			
	Paired difference mean (95% CI)	Change (%)	Effect size (95% CI)	t	Paired difference mean (95% CI)	Change (%)	Effect size (95% CI)	t	Paired difference mean (95% CI)	Change (%)	Effect size (95% CI)	t	Paired difference mean (95% CI)		Change (%)	Effect size (95% CI)	t	Paired difference mean (95% CI)		Change (%)	Effect size (95% CI)	t
Male	-0.01 (-0.08, 0.07)	0.04%↓	-0.01 (-0.09, 0.07)	-0.21	0.12 (0.08, 0.16)	0.55%↑	0.11(0.07, 0.16)	5.46^***^	0.26 (0.15, 0.37)	1.03%↑	0.17(0.10, 0.25)	4.58^***^	0.38 (0.24, 0.51)		1.31%↑	0.21(0.13, 0.28)	5.47^***^	0.17 (0.13, 0.20)		0.72%↑	0.13(0.10, 0.16)	8.57^***^
Female	0.22 (0.14, 0.30)	1.20%↑	0.26 (0.16, 0.35)	5.36^***^	0.33 (0.21, 0.45)	1.58%↑	0.16(0.10, 0.21)	5.42^***^	0.47 (0.27, 0.68)	1.96%↑	0.37(0.21, 0.53)	4.61^***^	0.23 (-0.16, 0.61)		0.80%↑	0.11(-0.08, 0.30)	1.15	0.31 (0.23, 0.40)		1.49%↑	0.17(0.12, 0.21)	7.37^***^
Total	0.09 (0.03, 0.14)	0.48%↑	0.10 (0.04, 0.16)	3.17^**^	0.19 (0.14, 0.24)	0.90%↑	0.13(0.09, 0.16)	7.53^***^	0.30 (0.20, 0.40_	1.20%↑	0.21(0.14, 0.27)	6.00^***^	0.36 (0.23, 0.49)		1.25%↑	0.19(0.12, 0.26)	5.48^***^	0.21 (0.18, 0.25)		0.94% ↑	0.14(0.12, 0.17)	11.27^***^

**p<0.01; ***p<0.001; ↑: increase; ↓: decrease.

## 4 Discussion

By conducting three repeated tests each year from 2019 to 2020, we revealed the BMI changes of 6,156 Chinese students from Tsinghua University, concluding the whole phase of pre-lockdown, during lockdown and post-lockdown. While a remarkable increase in BMI was observed across genders during both the lockdown and post-lockdown periods, the increase exhibited variations in distinct sexual and weight groups. To our best knowledge, this is the first study that estimates the impact of the COVID-19 lockdown by conducting a large sample to follow up a three-year consecutive cohort study using objectively measurement from 2019 to 2021.

COVID-19 lockdown had a significant negative impact on Chinese college students’ BMI (30–34). According to the overall data in this study, all participants’ BMI exhibited a significant increase (4.21%, p<.001) during the lockdown. This finding is consistent with previous research that claimed people’s BMI would become higher during the COVID-19 lockdown (14, 35–37). One plausible explanation for this increase could be that the lockdown increased people’s unhealthy food intake, changed their lifestyle, and limited their access to exercise resources thus reducing their engagement in physical activities (38–44). Furthermore, the implementation of lockdown measures had a negative influence on people’s mental health, which was also proved to have correlations with their weight gain (21). Additionally, predominant mild/asymptomatic SARS-CoV-2 infection also was showed to be associated with increase in BMI (45).

In the post-lockdown test, participants continued to exhibit a notable increase in their BMI (0.94%, p<0.001). However, the growth rate had a prominent reduction compared with the last assessment. This finding is consistent with previous Japanese research that indicated BMI gains decreased in different weight groups after the lockdown (46). One possible explanation concludes the uniqueness of the subjects in our study. Previous studies ahead of the pandemic had proved that college students experience weight gain in the 4 years of campus life (47–49). One year after the lockdown, the impact of the pandemic on the weight gain was reduced, thus BMI changes of students returned to the normal growth pattern.

However, our findings inconsistent with previous research that showed no significant change in college students’ BMI after the COVID-19 lockdown (24). One plausible explanation for the inconsistent is the difference in the sample size. Our study assembled 6156 college students as the subjects while in Masud et al. reported that there were 233 college students enrolled.

Our study also found that during the lockdown, people with higher BMI showed the slowest increase. The obese and overweight group’s growth rate from 2019 to 2020 is smaller than the normal and underweight group. This finding is consistent with a previous study that explored the changes in the physical index of Chinese college students (50). It demonstrated that there existed no statistically significant difference among students with BMI over 24, but individuals with BMI<24 exhibited an increase. However, our finding is different from one research in Switzerland, whose data showed the obese group had a higher increase than the control group (14). One possible explanation is the subject’s difference. Our study only aimed to investigate college students, while the study in Switzerland concluded both children and adolescents. Furthermore, our outcome indicated that after the lockdown, people with higher BMI showed a more prominent increase. In the post-lockdown period from 2020 to 2021, the obese and overweight group’s BMI growth rate was greater than the normal and underweight group, which were 1.25%, 1,2%, 0.9%, and 0.48%, respectively. Further research is needed to explore the BMI fluctuations across varying weight groups during both the lockdown and post-lockdown periods.

In terms of gender, this study demonstrated that during the lockdown, males presented a greater BMI growth rate than females, which are 4.74% and 2.95%, respectively. This finding is consistent with a previous study in Italy (51). However, after the lockdown, females exhibited a greater BMI growth rate than males, which were 1.49% and 0.72%, respectively. This phenomenon may be because girls tend to pay more attention to body shapes, so the possibility of greater weight gain in females during the pandemic is less than in males. On the other hand, boys are more easily affected by the restrictions of public exercise resources, thus males possess a higher possibility of greater weight change before and after the lockdown.

Interestingly, our study also found variations in BMI among different weight groups. During the lockdown, the underweight group exhibited the greatest growth rate in both genders (5.92% for males and 4.35% for females, p<0.001), while overweight group (1.28%, p<0.001) and obese group (2.95%, p<0.001) had the modest BMI growth increase in females and males, respectively. After the lockdown, the overweight (1.96%, p<0.01) and obese group (1.31, p<0.001) are the groups that presented a modest increase in females and males, respectively. These findings revealed the varied impact of the pandemic lockdown on people upon their different BMI baseline and gender.

This study possessed three strengths. First, it tracked the same samples’ changes in three consecutive years, including the whole phase of pre-COVID-19, during COVID-19 and post-COVID-19. By conducting the three-year follow-up study, this research examined the whole progression of the pandemic and explored the impact of the COVID-19 lockdown on people. Moreover, the sample size of this study is 6,156, which is more abundant than the previous studies in this field. The large sample is capable of diminishing the bias through the study and offering a more reliable result. Last but not least, by doing the subgroup analysis, this study can furnish a more specific comprehension of the pandemic lockdown’s impact on college students with different BMI baselines and gender.

Limitations also exist in this research. First, the percentage of the samples’ gender has an inherent imbalance. The disparity of gender ratio may induce research bias in this study. Further studies should incorporate this aspect into consideration and recruit samples with a balanced number of genders. Furthermore, this study evaluated the impact of COVID-19 on students’ BMI in one university in China, which has less representativeness. Research should be replicated at other universities in China to show some further results. In addition, this study only concludes objective indicators rather than other subjective indicators such as body image. Future study should take both objective and subjective indicators into consideration. Moreover, the “during lockdown test” was conducted several weeks after the lockdown restrictions ended. Though it’s best likely to reflect the BMI changes of participants, some bias still may exist. Besides, this study didn’t collect participants’ comorbid conditions nor SARS-CoV-2 infection data. These are significant confounder and future studies should refine the research design, control the confounders and figure out a clearer relationship between pandemic lockdown and BMI among college students. Last but not least, this study design only included students from 18 to 22. More age groups of people can be included in future studies to see the impact of the pandemic on various types of populations.

## 5 Conclusion

This study estimated the impact of the COVID-19 pandemic lockdown on 6156 Chinese students by conducting a three-year follow-up cohort study from 2019 to 2021. The outcome demonstrated that BMI in Chinese college students exhibited a notable increase for either gender during the period. Furthermore, during lockdown, people with higher BMI have the potential to have a diminished BMI growth rate, whereas the overall BMI growth rate in males is greater than in females. After the lockdown, people with higher BMI tend to possess a greater BMI growth rate, whereas the overall BMI growth rate in females is greater than in males. Public policy action is needed to increase the level of physical activity of Chinese college students and take action to improve students’ physical fitness performance after the lockdown.

## Data availability statement

The raw data supporting the conclusions of this article will be made available by the authors, without undue reservation.

## Ethics statement

This research was approved by the ethics committee of the university (IRB #2012534001). The studies were conducted in accordance with the local legislation and institutional requirements. The participants provided their written informed consent to participate in this study. Written informed consent was obtained from the individual(s) for the publication of any potentially identifiable images or data included in this article.

## Author contributions

HL: Writing – review & editing, Writing – original draft. YS: Writing – review & editing, Supervision, Formal analysis. YW: Writing – review & editing, Methodology, Investigation, Data curation. XF: Writing – review & editing, Supervision, Methodology, Investigation, Formal analysis. CL: Writing – review & editing, Resources. JP: Writing – review & editing, Resources. HY: Writing – review & editing, Visualization, Validation, Supervision, Resources, Project administration, Methodology, Investigation, Funding acquisition, Formal analysis, Conceptualization.
